# Sleeping Sickness

**Published:** 1921-01-22

**Authors:** 


### Sleeping Sickness.

There seems to be some public anxiety as to the prevalence of what is called "sleeping sickness" in this country. But the latest available return shows only about one case per million inhabitants of England and Wales. It is stated at the Ministry of Health that a number of cases have been notified during the past week, but to describe the condition as an epidemic, as it has already been described in some quarters, is altogether misleading and likely to cause unnecessary alarm. Cases as soon as they were reported have been investigated by the medical officers of the Ministry, who state that they have not detected any abnormal features in the outbreak nor any large increase in the number of cases usually reported at this time of the year. It is also of importance to remember that a number of cases hitherto reported as infantile paralysis have recently been diagnosed as encephalitis lethargica, thus swelling the total of persons reported to be suffering from the disease. The Ministry is unable to confirm the report that from 20 to 30 per cent. of the authenticated cases prove fatal. In Paris last spring there were 1,600 cases, with a high rate of mortality, but the cases in this country during the past few weeks have been of a mild type, yielding generally to medical treatment.

